# High-utilizing Crohn's disease patients under psychosomatic therapy*

**DOI:** 10.1186/1751-0759-2-18

**Published:** 2008-10-13

**Authors:** Hans-Christian Deter, Jörn von Wietersheim, Günther Jantschek, Friederike Burgdorf, Brigitta Blum, Wolfram Keller, the German Study Group on Psychosocial Intervention in Crohn's Disease

**Affiliations:** 1 Department of Psychosomatics and Psychotherapy, Charité Campus Benjamin Franklin, Berlin, Germany; 2 Department of Psychosomatic Medicine and Psychotherapy, Medical University Lübeck, Germany; 3 Medical Clinic I Gastroenterology, Charité Campus Benjamin Franklin, Berlin, Germany

## Abstract

**Objective:**

Few studies have been published on health care utilization in Crohn's disease and the influence of psychological treatment on high utilizers.

**Methods:**

The present sub study of a prospective multi center investigation conducted in 87 of 488 consecutive Crohn's disease (CD) patients was designed to investigate the influence of the course of Crohn's disease on health care utilization (hospital days (HD) and sick leave days (SLD) collected by German insurance companies) and to examine the conditions of high-utilizing patients. Predictors of health care utilization should be selected. Based on a standardized somatic treatment, high health care utilizing patients of the psychotherapy and control groups should be compared before and after a one-year treatment.

**Results:**

Multivariate regression analysis identified disease activity at randomization as an important predictor of the clinical course (r^2 ^= 0.28, p < 0.01). Health care utilization correlated with duration of disease (p < 0.04), but the model was not significant (r^2 ^= 0.15, p = 0.09). The patients' level of anxiety, depression and lack of control at randomization predicted their health-related quality of life at the end of the study (r^2 ^= 0.51, p < 0.00001). Interestingly, steroid intake and depression (t1) predicted the combined outcome measure (clinical course, HRQL, health care utilization) of Crohn's disease at the end of the study (r^2 ^= 0.22, p < 0.001).

Among high utilizers, a significantly greater drop in HD (p < 0.03) and in mean in SLD were found in the treatment compared to the control group.

**Conclusion:**

The course of Crohn's disease is influenced by psychological as well as somatic factors; especially depression seems important here. A significant drop of health care utilization demonstrates the benefit of psychological treatment in the subgroup of high-utilizing CD patients. Further studies are needed to replicate the findings of the clinical outcome in this CD subgroup.

## Introduction

Thought to influence perceived health in inflammatory bowel disease (IBD), patients' care-seeking behavior has aroused increasing medical and economic interest, especially since high users of medical care have turned out to be a serious therapeutic problem [1]. Due to the chronic course of their disease with frequent relapses, IBD patients tend to have more medical care utilization (doctor visits and hospital days per year) than patients with other gastrointestinal diseases [2]. Physical conditions as well as psychosocial factors are thought to influence the health status perception of IBD patients and thus also their medical care utilization [4]. The structure of the health care system in a region or country may also affect medical health care utilization.

Due to its chronicity, Crohn's disease (CD) not only leads to physical complaints but also causes many patients to develop psychological symptoms [5-10] that may influence their care-seeking behavior. Physical complaints and psychological factors like coping style may also influences the health-related quality of life in CD patients [11-14]. There is evidence that psychosocial factors accelerate the progression of Crohn's disease [15,16], and the individual health status of CD patients seems to be more closely related to psychological factors than to somatic ones [4]. The medically defined severity of illness failed to correlate significantly with variables of health care utilization in Crohn's disease patients [17].

This paper aims at reevaluating these findings using data from a previously reported randomized trial, which have focused on the somatic course of disease [18] and on psychological outcome [19]: it investigates whether factors relating to the bio psychosocial situation of Crohn's disease patients correlate with their health care utilization and whether high health care utilizers benefit from receiving psychological treatment in combination with standardized medical therapy [19]. Psychotherapeutic interventions and educational programs have been positively assessed for their effectiveness in controlled studies with both good [20-23] and discouraging results [24,25], but few controlled studies have been conducted on health care utilization in Crohn's disease [26,27].

We investigated the following hypotheses:

1. Psychological and clinical factors predict health care utilization, the somatic course of disease and the health-related quality of life in CD patients.

2. Psychological treatment reduces health care utilization in high-utilizing CD patients.

## Methods

### Patients

#### Recruitment and screening procedure

During the two-year recruitment period, all consecutive CD patients from the four participating centers were documented by recording their anamnestic and underlying somatic clinical data. The criteria for inclusion in the study were: Confirmed diagnosis, age between 18 and 55 years, at least one active disease episode (defined as requiring drug treatment) in the last two years, informed consent to participate and to be randomized in a psychotherapy or non-psychotherapy group. The exclusion criteria were: psychotherapy or resection for Crohn's disease within the last two years and no further relapse thereafter, ongoing immunosuppressive therapy or resection in the near future, and colostomy or ileostomy.

Due to the restrictive inclusion and/or exclusion criteria, only 108 of 488 patients were randomized in the "PICD" study: 37 were randomized to the control group and 71 to the psychotherapy group. The largest group of non-participants comprised patients without any relapse in the two years prior to the basic documentation. Comparison of the included and excluded patients disclosed no significant differences in the distribution of sex, involvement pattern, or disease duration. As the inclusion/exclusion criteria indicate, the participants were on average younger than the non participants, and fewer of them had undergone previous resections [18].

#### Dropouts due to non-fulfillment of the health care utilization criteria

twenty-nine of the 108 patients (26.9%) who met the inclusion and exclusion criteria could not be evaluated for health care utilization. They failed to obtain the data from the German insurance companies within the collecting period. In further ten patients more then 10% missing data caused exclusion from the study. The patients who dropped out were on average younger, and fewer of them had undergone previous resection. Comparison with the eligible patients revealed no differences in the distribution of the involvement pattern, sex, or disease activity at the time of randomization.

To carry out the following evaluation (conduct the regression analysis and calculate effects in high-utilizing patients), we took the 69 randomized CD patients and completed in 9 other randomized patients the missing values in the data sets by means. 9 patients of 380 CD patients were also included. In this study they had preferred psychotherapy and were therefore not randomized, but they received the same psychological treatment and took part in all examinations of the study.

Patients were clinically examined at baseline and every three months during the two years of the study. Psychological examinations took place at baseline and after 12, 18 and 24 months. The same standardized drug treatment was provided in both groups (see below). All patients in the intervention group were entered into a psychological treatment program (see below). Patients in the control group received only the eight control examinations. The study was approved by the local ethics committee.

### Assessment of health care utilization

Data on hospital days (HD, n = 87) and sick leave days (SLD, n = 56) were collected for four years from the different German health insurance companies with the informed consent of the patients (SLD data were not available for housewives, students or unemployed patients). We were thus able to include data from two years of health outcome before randomization, one year after randomization during psychological treatment, and one year of follow-up. Analyses of costs (drugs, doctor visits, etc.) were planned but not performed due to insufficient data sets.

High utilizers (HU) of health care were characterized as follows: patients above the median HD during the two-year study period (n = 41; 28 in the psychotherapy group and 13 in the control group). The most important parameters like age, gender, prior resections and active disease at randomization were evenly balanced between the two treatment groups (Table 1). The same holds true for the socio demographic data: the HU treatment and HU control group did not differ in their family status, partnership, children, or education level in this sub analysis of a nonrandomized sample.

**Table 1:** Health care utilization – patients studied and influencing factors

		Health care utilization study		High-utilizing CD		Patients (n = 41)		
		total (n = 87)		Psychotherapy group (n = 28)		Control group (n = 13)		p
		n	%	n	%	n	%	
Male		33	37.9	15	37.5	13	54.2	n. s.
female		54	62.1	25	62.5	11	45.8	

age (years)	< 30	46	52.9	18	45	15	62.5	n. s.
	< 30	41	47.1	22	55	9	37.5	

previous	no	60	69	30	75	15	62.5	n. s.
resection	yes	27	31	10	25	9	37.5	

episode	no	54	62.1	14	35	10	41.7	n. s.
	yes	33	37.9	26	65	14	58.3	

involvement pattern								
only small intestine		12	14.9	6	15	4	16.7	n.s.
only large intestine		11	12.6	9	22.5	1	4.2	
small and large intestine		63	72.4	25	62.5	19	79.2	

		m	s	m	s	m	s	

disease duration (months)		64.3	62.8	65.4	67.7	64.4	44.0	n. s.

Crohn's disease activity index (Best)		153.7	98.1	143.4	93.4	168.3	98.7	n. s.

### Gastroenterological Assessment

Once included in the study, patients were subjected to the following examinations: complete history, clinical and laboratory examinations, colonoscopy, esophagogastroduodenoscopy, X-ray of the small intestine, and CDAI calculations [28]. The CDAI was also recorded during all follow-up examinations, and it was used to decide what drug treatment was necessary. Each patient's course was documented for two years. Somatic data were recorded every three months during remission and once a week during acute attacks.

### Psychosocial Assessment

The patients' psychosocial status was determined [18] on the basis of their depression self-ratings (Beck's Depression Inventory: BDI [29]), trait anxiety (STAI-X2 [30]), health-related quality of life (HRQL, 31), and coping behavior (CQ [32]).

### Combined measurement of health care utilization, the HRQL, and the somatic course of disease

We defined three outcome measurements (each on a 6-step evaluation scale from higher to lower values) to analyze clinically meaningful fields of the disease:

#### Health care utilization

six groups with utilization differences in HD and SLD during the two-year study period: 0 HD and 0 SLD = 0 (n = 18); 0 HD, 1–40 SLD = 1 (n = 18); 0 HD, >40 SLD = 2 (n = 9); 1–14 HD, 8–71 SLD = 3 (n = 9); 15–30 HD, 88–184 SLD = 4 (n = 4); >30 HD, 121–331 SLD = 5 (n = 6).

#### Health-related quality of life

six groups with combined mean values in the HRQL sum score [26]: six (t 18: m 60.4, sd 20.5) and twelve months (t 24: m 66.4, sd 20.3) after psychotherapeutic treatment.

#### Somatic course of Crohn's disease

six different groups after sub ranking the main outcome group (blinded estimation of each case): episode-free course = 0; course with episodes and response to corticoid therapy = 1–3; course with episodes and failure of standard therapy with corticoids = 4; course with surgery for intractable inflammatory activity = 5 [19].

This enabled a combined outcome measurement of three areas representing biological, psychological and health care aspects of the disease.

### Treatment

#### Drug treatment

Based on the study protocol of the European Cooperative Crohn's Disease Study/ECCDS [33], we used a fixed dosing scheme for administering corticosteroids during acute episodes: 60 mg of prednisolone daily as the initial dose was followed by weekly reductions to 40 mg, 30 mg, 25 mg, 20 mg, and 15 mg. Patients were given 10 mg/day from week 7 to 19 and 10 mg every other day from week 20 to 28. Sulfasalazine was allowed in patients with colonic Crohn's disease, 5-ASA in all cases. If remission or a significant reduction of CDAI [28] was not achieved after 6 weeks of drug treatment, the same scheme was repeated, beginning with 60 mg of prednisolone. No drug treatment was given during remission of the disease.

#### Psychological treatment

For the intervention group, all participating centers provided basic short-term psychodynamic psychotherapy (20 hours) and a relaxation treatment program (10 autogenic training sessions). The total length of psychotherapy was not to exceed one year. The mean duration was 47.0 weeks (SD 31.2) for therapy across the four study centers, 26.2 weeks (SD 20.5) for the total number of verbal therapy sessions, and 17.6 weeks (SD 10.4) for the mean number of relaxation therapy sessions.

The aim of verbal psychotherapy was to provide health education and health-promoting behaviors, to give patients greater responsibility and control over their treatment, and to improve their coping skills and adjustment to the disease. Another aim was to alleviate possible disease-related psychological distress and maladaptive interpersonal patterns. Although no manual was used, the psychotherapy provided was based on the principles of psychodynamic psychotherapy and was standardized within the study centers [18].

### Statistical analysis

Calculation of correlations (Spearman) and multivariate regression analysis were done with the whole sample of 87 CD patients to detect factors influencing health care utilization.

Therapeutic results were analyzed only in high-utilizing Crohn's disease patients. Patient selection and the homogeneity of the two therapy groups were evaluated with respect to important somatic and psychosocial parameters. The two HU treatment groups were compared with regard to their overall HD and SLD scores assessed two years before randomization (divided by two) and at the one-year follow-up after psychological treatment.

All group comparisons were performed with the Mann-Whitney U-test or the t-test for continuous or ordinal variables and Fisher's exact test for categorial variables. The alpha errors of the tests were adjusted according to Bonferroni-Holm in order ensure an overall significance level of α = 0.05. All calculations were performed using the Statistical Analysis System (SAS, SAS Institute, Cary, NC, USA).

## Results

Subjects in the high-utilizer study (n = 87) had a mean age of 31.3 years (sd 30.0) and showed female preponderance (62.1%). An acute episode of disease activity occurred in 37.9% of these patients at the beginning of the study, and 31 % had a history of bowel resections. Local findings involved the ileum and colon in 72.4% of the patients, the colon in 12.6%, and the ileum in 14.9% (Table 1).

On the BDI depression scale, this sample had a mean score of 11.5 (sd 8.1), which is below the range of mild depression. The mean STAI anxiety value was 41.5 (sd 10.7) which ranges between normal controls and a clinical population. The same was also true for the HRQL values (m 70.0, sd 20.0).

According to data collected from the German insurance companies, the examined 87 subjects had 24.4 HD (sd 32.3) and 92.6 SLD (sd 83.7) at the end of the two-year study period. It was interesting to note that the health care utilization (HCU) data collected from the patients every three months in a personal examination did not correlate very well with the data obtained from the insurance companies (IC). The IC data for HD correlated in only 62.5% before and 63.3% after randomization. The correlation for SLD was 54.5% before and 57.6% after randomization.

In a first step, we calculated both HD and SLD with important anamnestic and psychological variables: gender (the female gender was a strong predictor for the number of HD before (.32, p < 0.05) and after randomization (.25, p < 0.05)), the Crohn's activity index (CDAI, .35, p < 0.05), and the scale of cognitive diversity (CQ scale 1; -.32, p < 0.05) correlated with the number of SLD before randomization.

### Calculation of a combined outcome measure in health care utilization, the clinical course, and the HRQL

To combine different biological, psychological and HCU aspects in the course of disease, we created a common measurement of HCU with HD and SLD during the two-year study period. Using this instrument to rate outcome on a scale of 0 – 5, the course of disease within the two-year study period was 3.26 (sd 1.62) for health care utilization, 2.90 (sd 1.47) for the HRQL, and 2.39 (sd 1.57) for the clinical course of the disease. The sum score was 8.25 (sd 3.27)

The three outcome measurements in the course of the study did not show a very high correlation (significant only for the clinical course and the HRQL at .30, p < 0.05). However, we found correlations with interesting psychological and clinical factors in each of the three dimensions. In the univariate analysis, the combined health care utilization measure correlated most closely with the BDI depression scale, the steroid intake, and the number of SLD at t 1. The disease activity (CDAI), steroid intake and depression at t1 predicted the somatic course of disease. The HRQL showed the highest correlation with anxiety, depression, lack of behavior control, SLD in the years before randomization, and the duration of disease (Table 2a).

**Table 2:** Predictors of Crohn's disease and combined psychosomatic outcome measure in 87 CD patients within two years

a) Univariate correlation				
	*outcome of Crohn's disease (worse)*			
predictors (t_0_)	health care utilization	HRQL	clinical course	sum score
CDAI	_•_145	_•_040	_•_470***	_•_324*
steroid intake	_•_291*	_•_025	_•_352**	_•_306*
disease duration	_•_129	_•_226*	_•_01	_•_059
sick leave at randomization	_•_288*	_•_021	_•_315*	_•_354**
depression (BDI)	.587***	_•_306*	_•_384**	_•_248
anxiety (STAI)	_•_101	_•_655***	_•_255	_•_349*
behavior control (FK 2)	-_•_162	-_•_230*	-_•_069	-_•_043

b) Regression analysis				
Health care utilization (HCU, high)				
	Model: r^2 ^= 0.15	F = 2.32	p = 0.09	

		β	T	p
1. Disease duration		0.92	2.1	0.04

Health-related quality of life (HRQL, low)				
	Model: r^2 ^= 0.51	F = 15.5	p = 0.00001	

		β	T	p
1. Anxiety (STAI)		-0.45	-3.9	0.0003
2. Behavior control (FK 2)		0.22	2.0	0.05

Clinical course of disease (CCD, worse)				
	Model: r^2 ^= 0.28	F = 6.16	p = 0.006	

		β	T	p
1. CDAI (t_0_)		-0.43	-2.9	0.007

HR sum score: (HCU, HRQL, CCD)				
	Model: r^2 ^= 0.22	F = 7.9	p = 0.0009	

		β	T	p
1. steroid intake		0.31	2.6	0.01
2. depression (BDI)		0.30	2.5	0.02

* p < 0.05 ** p < 0.01 *** p < 0.001

In separate multiple regression analyses of the somatic, psychological and health care status, the disease duration was a significant predictor of health care utilization (combined measurement of HD and SLD), but the model was not significant (r^2 ^= 0.15, p = 0.09).

Multivariate regression analysis identified disease activity at randomization as an important predictor of the clinical course (r^2 ^= 0.28, p < 0.01). The patient's level of anxiety and lack of control at randomization predicted the health-related quality of life at the end of the study (r^2 ^= 0.51, p < 0.00001).

In a summarized regression analysis of all the above-mentioned parameters, steroid intake and depression (t1) proved to be highly significant predictors of the combined outcome measure (clinical course, HRQL, health care utilization) of Crohn's disease in the long run (r^2 ^= 0.22, p < 0.001, Table 2b).

### Effect of psychosomatic treatment in high utilizers

We created a group of 41 high-utilizing patients (HU) above the 50^th ^percentile of HD by median split (more than 17 HD within the two-year study period) and a group of low-utilizing patients (LU, <17 HD). The two groups did not differ significantly variables of influence like age, earlier resections or current episodes. However, the high utilizers showed a female preponderance (p < 0.05). Hospital days were 44.4 (sd 36.0) for HU and 4.9 (sd 6.26) for LU. Sick leave days were 129.3 (sd 89.1) for HU (n = 34) and 53.6 (sd 56.5) for LU (n = 32).

**Figure 1:**
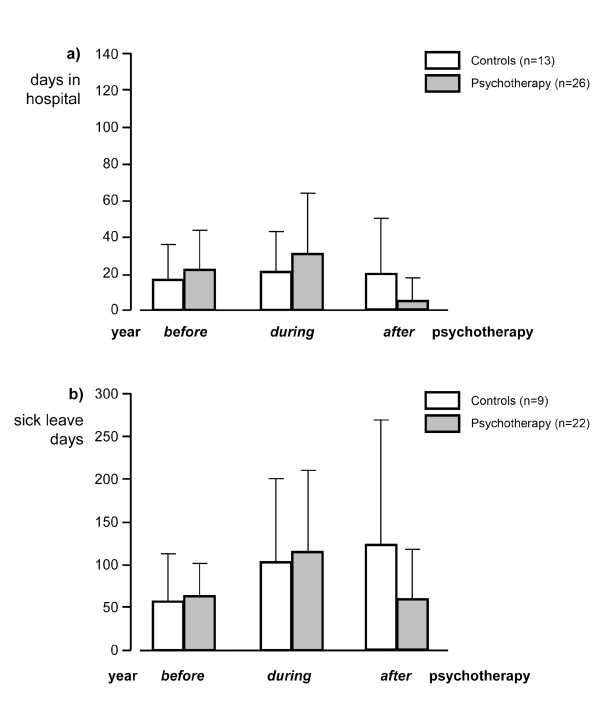
**High utilizer with Crohn's disease within three years before, during and after psychotherapy 1a) days in hospital (DH; Mann Whitney test p < 0.05) and 1b) sick leave days (SLD; Mann Whitney test p < 0.1).** SLD data were not available for house wives, students and unemployed patients. So the control group (n = 9) and the psychotherapy group (n = 22) showed a lower number of patients.

Calculating the treatment study results in 41 high-utilizing patients (28 in the psychotherapy group and 13 in the control group, Table 1) revealed a significant difference in HD (p < 0.0001) between the treatment group (24.2 (sd 20.8) to 5.7 (sd 13.4)) and controls (17.6 (sd 7.6) to 19.9 (sd 32.9)) in the year before randomization compared to the year of follow-up. The mean SLD decreased in the treatment group (67.9 (sd 40.0) to 57.8 (sd 81.4)) and increased in the control group (55.5 (sd 57.2) to 123.9 (sd 145.6)), but this was not significant, since the calculation involved only the reduced number of employed patients (19 therapy group; 8 control group, Fig. 1). Interestingly, the adherence of patients to the general practitioners did not change in the course of time, the number of visited doctors per year remaining nearly the same in both groups.

## Discussion

The present trial evaluated the influence of somatic and psychological factors on health care utilization in the course of Crohn's disease (CD) and the effect of a psychological intervention on high-utilizing CD patients. The somatic and psychosocial course of CD patients submitted to a psychological intervention has already been described [18,19]. Eighty-seven of 488 consecutive patients in the four participating centers were included in the protocol of the sub study evaluated here. Due to an ambitious study protocol, 75% of the screened patients were excluded [19]. High patient adherence was required in view of the many clinical and psychological examinations as well as the collection of health care data from German insurance companies. These patients were selected for showing high disease activity without requiring a surgical intervention. Moreover, there was a relatively high dropout rate in collecting the bio psychological and health care utilization data. Comparing the epidemiological data of the patients included with those of larger cohorts [34,35] shows a slightly higher mean age. This can be explained by the fact that our study only included patients over the age of 18. There were more women than men in our study, while the literature generally reports a balanced gender ratio [36]. The disease distribution along the intestinal tract in our study population is comparable to that reported in other studies [36,37]. The study protocol excluded patients seeking psychological treatment for psychosocial problems as well as those exhibiting a severe somatic course with psychological distress. Psychological data thus showed few psychological symptoms in our study patients. A low baseline level of psychosocial distress in IBD patients was also confirmed by Drossman et al. [5]. This is consistent with data indicating that, despite their symptoms, patients with IBD perceive their health-related quality of life as being quite good [10].

With the aim to present an appropriate outcome measure, it was very important to describe the somatic course of the disease over a two-year period. We used the ECCDS protocol [34] to develop a ranking scheme ranging from the best to the worst clinical course. This enabled very careful evaluation of the somatic outcome criterion in 87 patients.

After a six- and twelve-month follow-up, we also assessed the HRQL by a self-rating questionnaire and a well known psychological outcome measurement.

This study was the first to collect CD health care data from German insurance companies (with surprising results regarding the validity of clinical HCU data collection). We are thus able to present a validated third (HCU) outcome variable. We found a high rate of care-seeking behavior in our patient population. It seemed very helpful in this context that patients were also checked very carefully with regard to their somatic and psychological course. However, the longer collecting time and complex analyses of data are the cause of this study being completed much later than our first publication.

The predictor analyses according to the amount of health care utilization in the study population (combined measure of HD and SLD) detected steroid intake, sick leave status, depression, and gender as significant variables of the univariate analysis. This is in agreement with other studies [5,7,14,38]. However, all variables except disease duration failed to reach statistical significance in the multiple regression analysis, and the statistical model with r^2 ^= 0.15 was not significant. The findings in this analysis thus remain weak and unconvincing.

The HRQL in the course of disease could be predicted by psychological, anamnestic and HCU variables (anxiety, depression and coping skills as well as the disease duration and the number of sick leave days in the two years before examination). This is in agreement with other studies [11-15,39]. Anxiety and coping skills could be included in a highly significant statistical model with r^2 ^= 0.51.

Interestingly, the somatic course of disease could be predicted by variables of acute inflammation (CDAI, steroid intake), psychological indicators (depression), and variables of health care utilization (SLD). Regression analysis identified the CDAI as the only significant variable in a significant model (r^2 ^= 0.28).

By combining the three outcome measurements of health care utilization and the HRLQ as well as the clinical course, we identified depression and steroid intake at randomization as highly significant predictors of the course of illness over two years in a significant statistical model (r^2 ^= 0.22). Disease activity and depression are well-known predictors of the course in Crohn's disease patients [7,11,14].

The carefully selected outcome variables enabled us to examine the effectiveness of a psychological treatment in the high-utilizing sub sample. Since there is only a poor response concerning the somatic course of disease to psychological treatment in CD patients it seems important to analyze sub populations. The definition of high utilizers in this study were done by median split of the medical health care data. Whether this is useful in general future studies have to demonstrate. It seems interesting that health care utilization in terms of hospital days and sick leave days was much higher in this CD study group than in other studies [2,5,24,25]. Here we have to take into consideration the health care situation in Germany, in the study cities and their surrounding regions. However, the fact that women had much higher HD and SLD values than men suggests that factors beside the somatic course of illness are important here.

Forty-one high-utilizing patients showed significant differences in the course of HD and SLD (difference between the value one year before randomization and at the one-year follow-up) with an advantage for the intervention group. Thus the present study was able to demonstrate the effectiveness of psychological treatment in reducing the health care utilization of these high-utilizing Crohn's disease patients. This seems interesting, since the randomized study failed to support significant somatic or psychological improvement following the psychological intervention [18,19] but showed a significant decrease of HD in the treatment group [40]. It is noteworthy that the results of the randomized study also hold true for high-utilizing Crohn's disease patients [26]. We were thus able to show that a psychological treatment was benefitial for this CD subgroup of high and cost-intensive health care utilizers without worsening their adherence to the medical regimen. The psychological focus in treating a CD subgroup of patients to change their psychological status and health care utilization is new and has thus far only been used in this sample of CD patients. Moderating factors seem to be the patients' more effective disease self-management, better adherence and more security in illness behavior during disease crises. Depressive symptoms decreased in CD patients, who had formerly high levels of depression [19]. A longer waiting time before (or neglect of) bowel surgery may be another reason. Greater skill in coping with the illness or with stressful life events may lead to fewer psychic symptoms [18-20] and a better HRQL [1,14,15] as well as risk factors like dietary habits or smoking may influence the possibility of going to the hospital or going to work [5]. These aspects could only be partly detected by our psychological measurement (18), but we assume they are meaningful for the change in health care utilization in the psychological treatment group [26].

Several aspects and limitations should be taken into account when interpreting the present results. The many exclusion criteria in the original trial [19] led to an examination rate of 87 in this sub study with 488 screened CD patients from specialized GI university medical centers. 87 of these patients included in this health utilization study came from a randomized sample [69], an additional sample wanted psychotherapy [9] and a third group had a high rate of missing data [9] but could be accepted in this analysis. Patients with surgery or those without relapses within the two years before the trial were excluded. Few patients with psychic disturbances like depression, anxiety or a low HRQL were included in the study.

The treatment results relate to a high-level health care population without severe psychiatric co morbidity and were obtained over an observation period of four years. It is possible that the treatment effect on health care utilization would be greater if we examined CD populations with higher psychiatric co-morbidity.

The type and dose of applied psychological treatment in this study should also be taken into consideration: It seems helpful to spend a minimum amount of time communicating with CD patients [41] and to provide information and offer education programs [21,22], but an intensive psychological treatment program [24,25,34] that focuses on changing coping skills and illness behavior [15] may intensify common treatment effects in high-utilizing CD patients.

## Conclusion

In summary, health care utilization proved to correlate closely with the somatic course as well as with psychic and anamnestic variables of the disease. Inflammatory activity and depression were important predictors of the combined biological, psychological and health care utilization endpoint of Crohn's disease. Thus it seems useful to describe a subgroup of high-utilizing Crohn's disease patients. Interestingly, they proved likely to benefit from psychological treatment. Variables of health care utilization should be included in future therapy studies, especially those examining high-utilizing Crohn's disease patients.

## Competing interests

The authors declare that they have no competing interests.

## Authors' contributions

HCD coordinated the care utilization sub study of "PICD" and drafted the manuscript. JvW participated in the design of the study and collected data in the Lübeck center. GJ participated in the design of the study and was responsible for the somatic outcome evaluation in the "PICD"-study. FB performed the statistical analysis. BB coordinated the data management. WK participated in the design of the study conceptualized the therapeutic intervention and was one of the therapist. All authors read and approved the final manuscript.

## Notes

* Results of the German prospective multicenter trial: Psychosicial Intervention in Crohn's Disease ("PICD").

## References

[B1] Katon W, Korff Mv, Liu F, Bush T, Russo J, Lipscomb P, Wagner E (1992). A randomised trial of psychiatric consultation with distressed high utlilizas. Gen Hospital Psychiatry.

[B2] Verhoefs M, Sutherland L (1995). Outpatient health care utilization of patients with inflammatory bowel disease. Dig Dis.

[B3] Hay AR, Hay JW (1992). Inflammatory bowel disease: cost of-illness. J Clin Gastroenterol.

[B4] Drossman DA, Patrick DL, Mitchell CM, Zagami EA, Appelbaum MI (1989). Health-related quality of life in inflammatory bowel disease- functional status and patient worries and concerns. Dig Dis Sc.

[B5] Helzer JE, Chammas S, Norland CC, Stillings WA, Alpers DH (1984). A study of the association between Crohn's disease and psychiatric Illness. Gastroenterology.

[B6] Andrews H, Barczak P, Allan RN (1987). Psychiatric illness in patients with inflammatory bowel disease. Gut.

[B7] Deter HC, Rapf M, Gladisch R, Rohner R (1993). Psychodiagnostische Verlaufsuntersuchungen von Morbus-Crohn-Patienten während der internistischen Intensivbehandlung. Z Gastroenterol.

[B8] Fullwood A, Drossman DA (1995). The relationship of psychiatric Illness with gastrointestinal disease. Annu Rev Med.

[B9] Levenstein S, Li Z, Almer S, Barbosa A, Marquis P, Moser G, Sperber A, Toner B, Drossman DA (2001). Cross-cultural variation in disease-related concerns among patients with inflammatory bowel disease. Am J Gastroenterol.

[B10] Janke KH, Klump B, Gregor M, Meisner C, Haeuser W (2005). Determinants of life satisfaction in inflammatory bowel disease. Inflamm Bowel Dis.

[B11] Bernklev T, Jahnsen J, Aadland E, Sauar J, Schulz T, Lygren I, Henriksen M, Stray N, Kjellevold O, Vatn M, Moum B, IBSEN Study Group (2004). Health-related quality of life in patients with inflammatory bowel disease five years after the initial diagnosis. Scand J Gastroenterol.

[B12] Eijk I van der, Vlachonikolis IG, Munkholm P, Nijman J, Bernklev T, Politi P, Odes S, Tsianos EV, Stockbrügger RW, Russel MG, EC-IBD Study Group (2004). The role of quality of care in health-related quality of life in patients with IBD. Inflamm J Bowel Dis.

[B13] Mussell M, Bäcker U, Nagel N, Singer MV (2004). Predictors of disease-related concerns and other aspects of health-related quality of life in outpatients with inflammatory bowel disease. Eur J Gastroenterol Hepatol.

[B14] Zaag-Loonen H Van der, Grootenhuis MA, Last BF, Derkx HH (2004). Coping strategies and quality of life of adolescents with inflammatory bowel disease. Qual Life Res.

[B15] Mittermaier C, Dejaco C, Waldhoer T, Oefferlbauer-Ernst A, Miehsler W, Beier M, Tillinger W, Gangl A, Moser G (2004). Impact of depressive mood on relapse in patients with inflammatory bowel disease: a prospective 18-month follow-up study. Psychosom Med.

[B16] von-Wietersheim J, Köhler T, Feiereis H (1992). Relapse – precipitating life events and feelings in patients with inflammatory bowel disease. Psychother Psychosom.

[B17] Drossman D, Leserman J, Mitchel C (1991). Health status and health care use in persons with inflammatory bowel disease: a national sample. Dig Dis Sci.

[B18] Keller W, Pritsch M, von Wietersheim J, Scheib P, Osborn W, Balck F, Dilg R, Schmelz-Schumacher E, Doppl W, Jantschek G, Deter HC, The German Study Group on Psychosocial Intervention in Crohn's Disease (2004). Effect of psychotherapy and relaxation ion the psychosocial and somatic course of Crohn's disease: main results of the German Prospective Multicenter Psychotherapy Treatment study on Crohn's Disease. J Psychosom Res.

[B19] Jantschek G, Zeitz M, Pritsch M, Wirsching M, Studt HH, Rasenack J, Deter HC, Riecken EO, Feiereis H, Keller W, the German Study Group on psychosocial intervention in Crohn's disease (1998). Effect of Psychotherapy on the course of Crohn's disease. Scand J Gastroenterol.

[B20] Milne B, Joachim G, Niedhardt J (1986). A stress management programme for inflammatory bowel disease patients. J Adv Nurs.

[B21] Schwarz SP, Blanchard EB (1991). Evaluation of a psychological treatment for inflammatory bowel disease. Behav Res Ther.

[B22] Smith GD, Watson R, Roger D, McRorie E, Hurst N, Luman W, Palmer KR (2002). Impact of a nurse-led counseling service on quality of life in patients with inflammatory bowel disease. J Adv Nurs.

[B23] Elsenbruch S, Langhorst J, Popkirowa K, Mueller T, Luedtke R, Franken U, Paul A, Spahn G, Michalsen A, Janssen OE, Schedlowski M, Dobos GJ (2005). Effects of mind-body therapy on quality of life and neuroendocrine and cellular immune functions in patients with ulcerative colitis. Psychother Psychosom.

[B24] Maunder RG, Esplen MJ (2001). Supportive-expressive group psychotherapy for persons with inflammatory bowel disease. Can J Psychiatry.

[B25] Larsson K, Sundberg Hjelm M, Karlbom U, Nordin K, Anderberg UM, Lööf L (2003). A group-based patient education programme for high-anxiety patients with Crohn disease or ulcerative colitis. Scand J Gastroenterol.

[B26] Kennedy AP, Nelson E, Reeves D, Richardson G, Roberts C, Robinson A, Rogers AE, Sculpher M, Thompson DG (2004). A randomized controlled trial to assess the effectiveness and cost of a patient orientated self management approach to chronic inflammatory bowel disease. Gut.

[B27] Waters BM, Jensen L, Fedorak RN (2005). Effects of formal education for patients with inflammatory bowel disease: a randomized controlled trial. Can J Gastroenterol.

[B28] Best WR, Becktel JM, Singleton JW, Kern F (1976). Development of a Crohn's Disease Activity Index. Gastroenterology.

[B29] Beck AT, Ward CH, Medelson M, Mock F, Erbaugh F (1961). An inventory of measuring depression. Arch Gen Psychiat.

[B30] Spielberger CD, Gorsuch RL, Lushene RE (1970). Manual for the state-trait anxiety inventory.

[B31] Bullinger M, Verres R, Hasenbring M (Hrsg) (1989). Forschungsinstrumente zur Erfassung der Lebensqualität bei Krebs – ein Überblick. Psychosoziale Onkologie Jahrbuch der Medizinischen Psychologie.

[B32] Thompson SC (1981). Will it hurt less if I can control it? A complex answer for a simple question. Psychological Bulletin.

[B33] Malchow H, Ewe K, Brandes JW, Goebell H, Ehms H, Sommer H (1984). European cooperative Crohn's disease study (ECCDS): Results of drug treatment. Gastroenterology.

[B34] Lind E, Fausa O, Gjone E, Mogensen SB (1985). Crohn's disease. Treatment and outcome. Scand J Gastroenterol.

[B35] Hellers G (1979). Crohn's disease in Stockholm county 1955–1974. A study of epidemiology, results of surgical treatment and long-term prognosis. Acta Chir Scand Suppl.

[B36] Farmer RG, Whelan G, Fazio UW (1985). Long-term follow-up of patients with Crohn's disease. Gastroenterolgy.

[B37] Binder V (1988). Epidemiology, course and socio-economic influence of inflammatory bowel disease. Schweiz Med Wochenschr.

[B38] Seibeni S, Cortinovis I, Beretta L, Tatarella M, Ferraris L, Rondonotti E, Corbellini A, Bortoli A, Colombo E, alvisi C, Imperiali G, de Franchis R, Gruppo di Studio per le Malattie Inflammatorie Intestinali (2005). Gender and disease activity influence health-related quality of life in inflammatory bowel diseases. Hepatogastroenterology.

[B39] Mitchell CM, Guyatt G, Singer J (1988). Quality of life in patients with inflammatory bowel disease. J Clin Gastroenterol.

[B40] Deter HC, Jantschek G, Studt HH, Wirsching M, Feiereis H, Zeitz M (1997). Effectiveness of psychotherapeutic measures in Crohn's disease. Psychosomatic Medicine.

[B41] Husain A, Triadafilopoulos G (2004). Communicating with patients with inflammatory bowel disease. Inflamm Bowel Dis (United States).

